# Antiangiogenic, wound healing and antioxidant activity of *Cladosporium cladosporioides* (Endophytic Fungus) isolated from seaweed (*Sargassum wightii)*

**DOI:** 10.1080/21501203.2016.1263688

**Published:** 2016-12-14

**Authors:** Manjunath M. Hulikere, Chandrashekhar G. Joshi, D. Ananda, Jagadeesh Poyya, T. Nivya

**Affiliations:** Department of Biochemistry, Mangalore University, P.G.Centre, Chikka Aluvara, Kodagu, India

**Keywords:** *Cladosporium cladosporioides*, *Sargassum wightii*, angiogenesis, antioxidant activity, Polyphenols, CAM assay

## Abstract

Endophytic fungi from marine seaweeds are the less studied group of organisms with vast medical applications. The aim of the present study was to evaluate antioxidant, antiangiogenic as well as wound healing potential of the endophytic fungus isolated from the seaweed *Sargassum wightii*. The morphological characters and the rDNA internal transcribed spacer sequence analysis (BLAST search in Gen Bank database) was used for the identification of endophytic fungus. The antioxidant potential of the ethyl acetate extract of endophytic fungus was assessed by, 1,1-diphenyl-2-picryl-hydrazyl radical scavenging method. The fungal extract was also analysed for reducing power, total phenolic and flavonoid content. Antiangiogenic activity of the fungal extract was studied *in vitro* by inhibition of wound healing scratch assay and *in vivo* by Chick chorioallantoic membrane assay. The endophytic fungus was identified as *Cladosporium cladosporioides* (Gen Bank ID – KT384175). The ethyl acetate extract of *C. cladosporioides* showed a significant antioxidant and angiosuppressive activity. The ESI-LC-MS analysis of the extract revealed the presence of wide range of secondary metabolites. Results suggest that C. *cladosporioides* extract could be exploited as a potential source for angiogenic modulators.

## Introduction

Angiogenesis is a complex process of formation of new blood vessels from the existing ones. This process is necessary for normal physiology such as wound healing, tissue growth, regeneration, etc. (Hanahan and Weinberg 2011). Tumour cells take advantage of angiogenesis for metastasis leading to the death of cancer patients. In addition to cancer, angiogenesis is involved in neurodegeneration, myocardial infarction, psoriasis, etc. (Folkman 1990; Carmeliet and Jain 2011; Hanahan and Weinberg 2011). Of late, researchers are focusing their attention on the modulators of angiogenic processes; as they can be exploited in controlling the disorders that are mediated through angiogenesis. Till now many patients have been benefited from the regulators of angiogenic proteins. Ten drugs (modulators of angiogenesis) have been approved since 2004 to treat various types of cancers and much more are in clinical trials. However, most of these therapeutic molecules have low efficacy and high toxicity (Carmeliet and Jain 2011; Jain 2014). So, the demand for the antiangiogenic therapeutic molecules with high efficacy and low toxicity is escalating.

Natural products or their derivatives are used in traditional as well as in many modern medicines for treating various ailments. Nearly 50% of the drugs approved recently were based on natural products (Kingston 2011). Out of 175 anticancer drugs discovered since 1940–2006, 47% were derived from either natural products or by semi-synthesis using natural products (Newman and Cragg 2007).

Microorganisms associated with the marine creatures such as algae, sponges, corals, tunicates, fishes, etc. have been extensively studied for their natural product content (Stamatios et al. 2013; García Caballero et al. 2014). Lately, marine microbes, especially fungi are emerging as a source of novel bioactive secondary metabolites (Penesyan et al. 2010). The kingdom of fungi is one of the largest groups of organisms found widely distributed in nature. Marine fungi have the capability to synthesise novel molecules as they exist in special ecological niches (Bhatnagar and Kim 2010; Raj and Vignesh 2011).

Marine endophytic fungi are less studied group of microorganisms even though they have the capability to produce an array of secondary metabolites such as alkaloids, benzopyranones, chinones, cytochalasins, depsipeptides, enniatins, flavonoids, peptides, polyketones, phenols, quinols, terpenoids, etc. that are reported to have many pharmacological implications (Miller et al. 2012; Qadri et al. 2013).

Marine endophytes have been extensively studied for various therapeutic potentials. However to our best of knowledge, no report was available on the antiangiogenic activity of seaweed endophytes except our recent report on the antiangiogenic potential of endophyte (*Penicillium citrinum*) isolated from seaweed (Manjunath et al. 2016). The present study was under taken to study the antiangiogenic, wound healing and antioxidant activity of another endophytic fungus isolated from seaweed, *Sargassum wightii*.

## Materials and methods

### Collection of seaweed

*S. wightii* was collected from the coastal region of Kanyakumari (Latitude 8.0780°N; Longitude 77.5410°E), Tamil Nadu, India and brought to the laboratory in a polythene bag along with seawater. The seaweed was authenticated by Dr. Lipton, Emeritus Professor (UGC) at CMST, M.S. University, Rajakkamangalam, Tamil Nadu, India.

### Isolation and identification of endophytic fungi

Endophytic fungus was isolated from marine seaweed (*S. wightii)* according to the protocol of Ariffin et al. (2014). *S. wightii* was washed thoroughly with seawater to expel epiphytic microorganisms, followed by tap water and distilled water to remove the salts and other materials. Seaweed was surface sterilised with 70% ethyl alcohol for 1 min followed by immersion in 4% sodium hypochlorite solution for 3 min and 70% ethyl alcohol for a minute. The surface sterilised *S. wightii* was rinsed with distilled water and blot dried on sterile tissue paper. Finally, the sample was rinsed with sterile distilled water and cut aseptically into 1 cm long segments. The cut segments of *S. wightii* were placed on potato dextrose agar (PDA) containing chloramphenicol. The plates were incubated at 28°C until mycelia were observed. A pure culture of endophytic fungus was isolated, subcultured on PDA without any antibiotic and incubated for 30 days at 28°C prior to extraction.

### Molecular identification

Total DNA of seaweed endophyte was isolated and polymerase chain reaction (PCR) was carried out using ITS 1 forward primer and ITS 4 reverse primer. 100 ng of genomic DNA, 100 μM dNTPs, and 2.5U Taq DNA polymerase were mixed in a PCR thermocycler. PCR was performed with 35 cycles of denaturation at 96°C for 45 s, annealing at 50°C for 45 s, extension at 72°C for 45 s and the final extension at 72°C for 10 min. The amplified product was subjected to electrophoresis using 0.8% agarose gel. The amplified DNA was purified and sequenced using the same primers used for PCR. NCBI BLAST N program was used to compare the sequence available in Gen Bank.

### Preparation of fungal submerged fermentation broth

The endophytic fungal isolates were cultured on potato dextrose broth for 21 days at 25–28°C (Ariffin et al. 2014).

### Extraction of secondary metabolites from the endophytic fungal culture

Extraction of secondary metabolites was carried according to the procedure explained by Ariffin et al. (2014) with slight modification. The endophytic fungal mycelium was mixed with an equal volume of ethyl acetate (100%) and the mixture was blended with equal volume of ethyl acetate. The resulting homogenate was filtered and stored at −20°C.

### Determination of total flavonoids

Total flavonoid content was measured according to the method of Kosalec et al. (2004) with slight modifications. Standard quercetin and ethyl acetate extract of the endophytic fungus (100 µg/ml) was taken in different test tubes. Volume in each tube was made up to 4.0 ml with distilled water and 0.3 ml of 5% sodium nitrate solution. After 5 min, 0.3 ml of 10% aluminium chloride was added and incubated for 6 min at room temperature. About 2 ml of 1 M NaOH and 3.4 ml of distilled water were added to all the tubes and the absorbance was read at 570 nm.

### Determination of total phenolic content

Total phenolic content was measured according to the procedure of Singleton and Rossi (1965) with slight modifications. Standard gallic acid and fungal extract (100 µg/ml) were mixed with 0.5 ml of Folin–Ciocalteu reagent (1:1), 2.5 ml of sodium carbonate (20%) and 6.0 ml of distilled. The absorbance of the reaction mixture was measured at 760 nm.

### Free radical scavenging assay by DPPH method

DPPH free radical scavenging potential of the extract was determined using the protocol explained by Blois (1958) with slight modifications. The DPPH stock solution was prepared by 0.1 mM DPPH in 99% ethanol and standardised to 1.9 ± 0.02 OD at 517 nm. About 2 ml (0.1 mM) DPPH was added to fungal extract (1 mg/ml) and standard ascorbic acid (0.1 mg/ml) and incubated for 30 min mixture. The absorbance of the reaction mixture was read at 517 nm. Percentage of the DPPH radical scavenging was calculated using the formula.

Inhibition of DPPH radical (%)=[(control absorbance−extract absorbance)/(control absorbance)]×100

### Total reducing power

The Fe^3+^reducing power of the extract was determined by the method cited in Oyaizu (1986) with slight modification. Different concentrations (0–500 µg/ml) of the extract (0.5 ml) were mixed with 0.5 ml phosphate buffer (0.2 M, pH6.6) and 0.5 ml potassium hexacyanoferrate (1%), followed by incubation at 50°C in a water bath for 20 min. After incubation, 0.5 ml of TCA (10%) was added to terminate the reaction. The upper portion of the solution (1 ml) was mixed with 1 ml distilled water and 0.1 ml ferric chloride solution (0.1%). The reaction mixture was left for 10 min at room temperature and the absorbance was measured at 700 nm against an appropriate blank solution. All tests were performed in triplicates. A higher absorbance of the reaction mixture indicated greater reducing power.

### Chick chorioallantoic membrane (CAM assay)

The CAM assay was performed as described by Muslim et al. (2012). Fertilised eggs were purchased from hatcheries. The eggs were incubated at 37°C in 40–60% humidity for 96 h and the eggs were randomly divided into two groups. After 7 days, a window (1 × 1.5 cm^2^) was made in the shell to expose a part of the CAM. Different concentration of the fungal extract (100 µg) was loaded on to sterilised Whatmans filter paper No.1 disc (2 mm^2^) was applied to the CAM. After 24 h of incubation, the number of new vessels in the CAM around the paper disc was photographed with the help camera (Sony). CAM samples were fixed in 10% formalin, embedded in paraffin, sectioned and finally stained with hemotoxylin and eosin for histopathological evaluation.

### Wound healing assay

To evaluate the effect of the fungal extract on cell motility, a scratching assay of the MCF-7 cells was performed MCF-7 cells were seeded in the six-well plates at a density of 1 × 10^5^. Reference points near the “scratch” were marked and the plates were incubated at 37°C. Cells were grown in DMEM having 10% FBS to a confluent monolayer for 2 days, and were scraped by 200 µL pipette tips to create a straight line cell-free “scratch”. Each well was washed twice with PBS and treated with extract and the standard drug (1 mg/ml). Migration of cells in the “scratch” was photographed at the matching reference points with an inverted microscope at intervals of 12 h (0, 12 and 24 h). The images were analysed quantitatively by Micam software; the distances between the two edges of the scratch were measured at the reference points and analysed statistically (Liang et al. 2007).

The cell migration rate was calculated with mean width using following the formula.

$$\begin{array}{l} \text{Cellmigrationrate}\%=({\text{width}}_{\text{0h}}-{\text{width}}_{\text{xh}}){\text{/width}}_{\text{oh}}\\ \;\;\;\;\;\;\;\;\;\;\times 100\% \end{array}$$

### Statistical analysis

All values were expressed as Mean ± SD. Comparison between the control and sample were performed by analysis of variance with Tukey’s multiple comparison tests using Graph pad Prism v3.0 software.

## Results

### Isolation of endophytic fungi from marine seaweed *S. Wightii*

**Figure 1.**
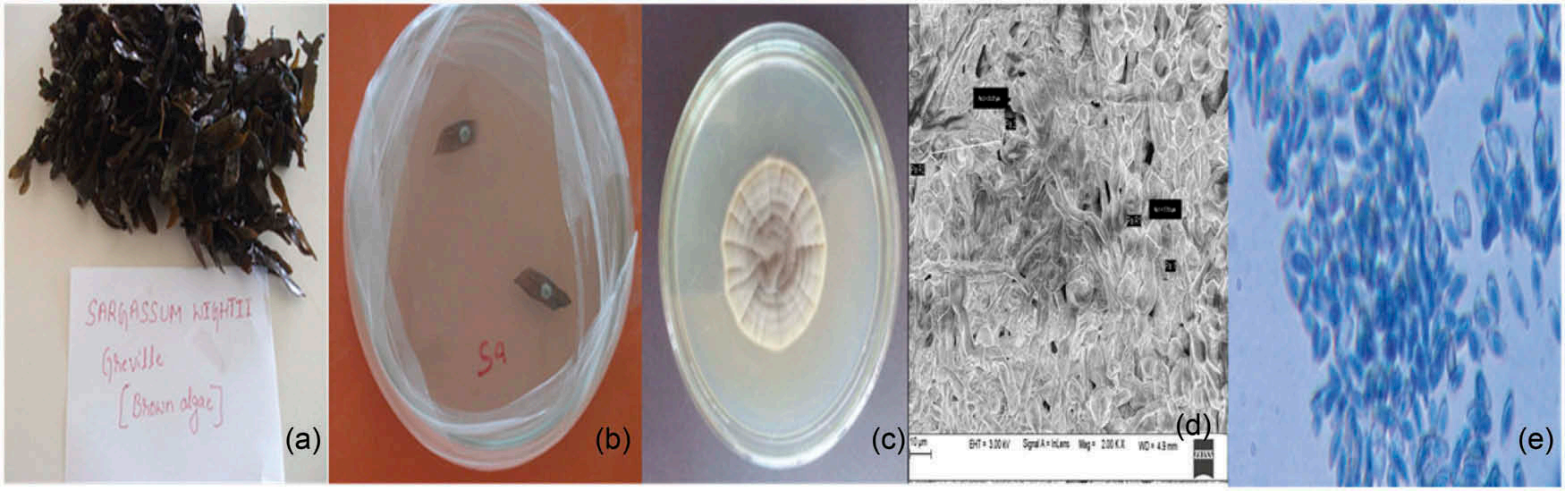
(a) Image of seaweed *S. wightii*. (b) *C. cladosporioides* grown from the segment of *S. wightii* on PDA. (c) Pure culture of *C. cladosporioides*. (d) FE SEM image of endophytic fungi, *C. cladosporioides* (e) Microscopic image of endophytic fungi, *C. cladosporioides.*

The aim of the present work was to isolate endophytic fungi from *S*. *wightii* and to study the antiangiogenic, wound healing and antioxidant activity. Out of the two endophytic fungi isolated from *S. wightii*, one was selected for further studies (Figure 1(a)). Microscopic observations and the morphological studies revealed the identity of endophytic fungus to be *Cladosporium sp*. (Figure 1(b)).

### Molecular identification

**Figure 2.**
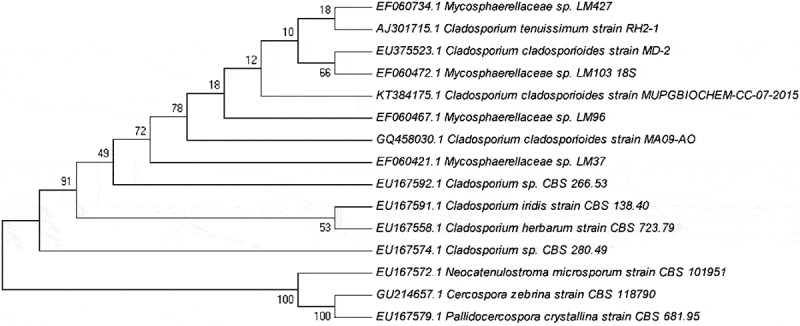
Phylogenetic tree of *C. cladosporioides.*

Molecular identification was achieved by comparing the rDNA internal transcribed spacer (ITS) region details with the established sequence. Organisms with 99% or more sequence similarity to be considered from the same species and between 93% and 98% similarity to be considered from the same genus over the organism in consideration to previously unidentified if the ITS Sequence similarity is below 93% (Bosshard et al. 2003). The endophyte had 99% similarity to *Cladosporium cladosporioides* (Figure 2). The identity of the organism was established as *C. cladosporioides* (Gen Bank ID – KT384175).

### Free radical scavenging activity by DPPH method

**Figure 3.**
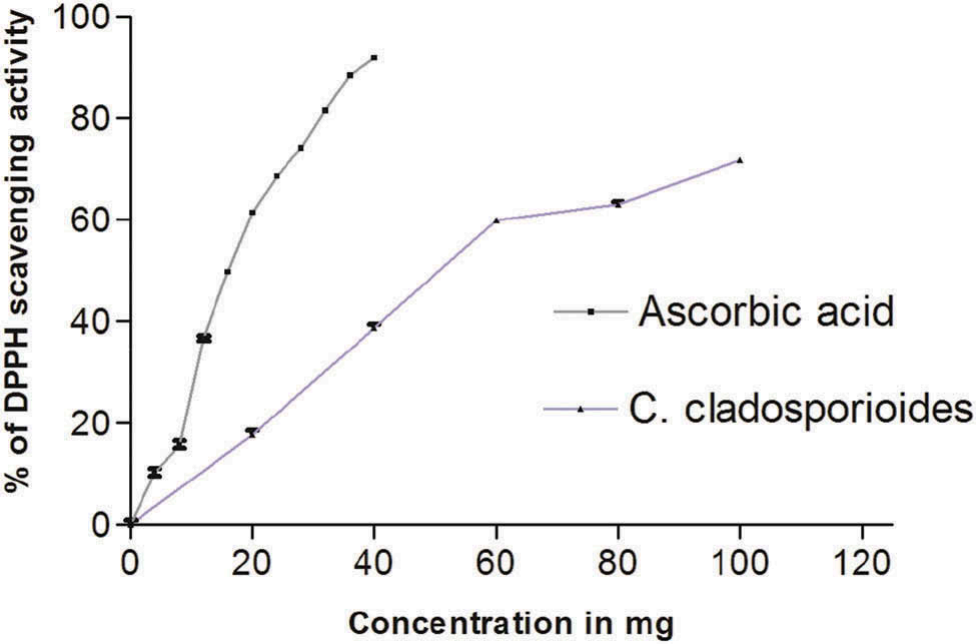
DPPH scavenging activity of ethyl acetate extract of *C. cladosporioides.*

The free radical scavenging activity of the ethyl acetate extract of *C. cladosporioides* was studied using DPPH method. Ascorbic acid was taken as standard. The endophytic *extract* showed a significant antioxidant activity and the activity of the extract was found to be concentration dependant. The antioxidant activity of ethyl acetate extract of the fungus was comparable to that of standard ascorbic acid as shown in (Figure 3).

The ethyl acetate extract was subjected to a quantitative analysis of total phenolics, flavonoids and reducing power assay showed the presence of 0.76 ± 0.04 mg/g total phenolics, 0.68 ± 0.02 mg/g of total flavonoids and 0.81 ± 0.03 mg/g of the reducing power, respectively.

### Antiangiogenesis activity by in vivo chick chorioallantoic membrane (CAM) assay

**Figure 4.**
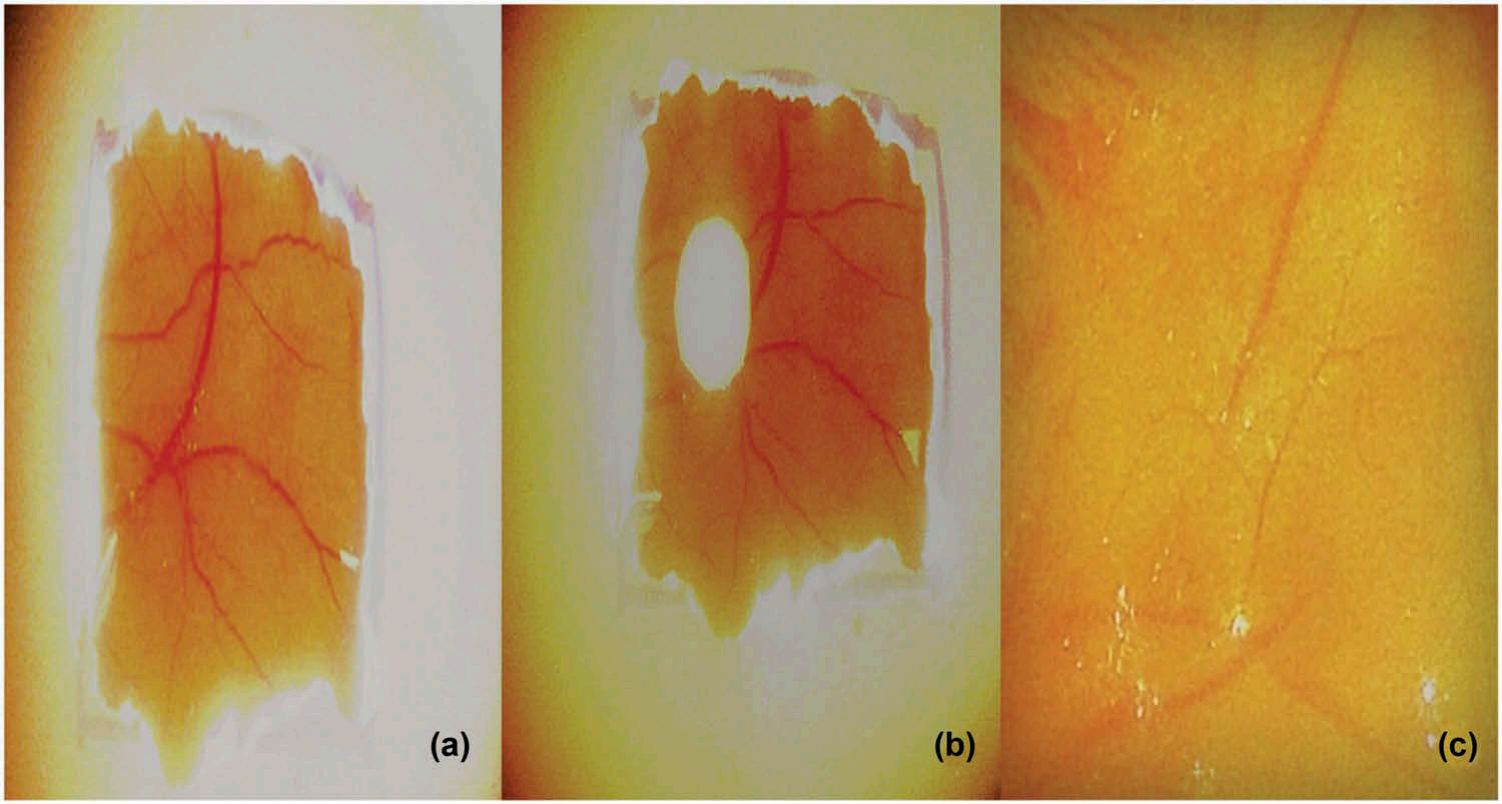
Angiogenesis in (a) Control. (b) *C. cladosporioides* extract on chorioallantoic membrane. (c) CAM with reduced blood vessels.

**Figure 5.**
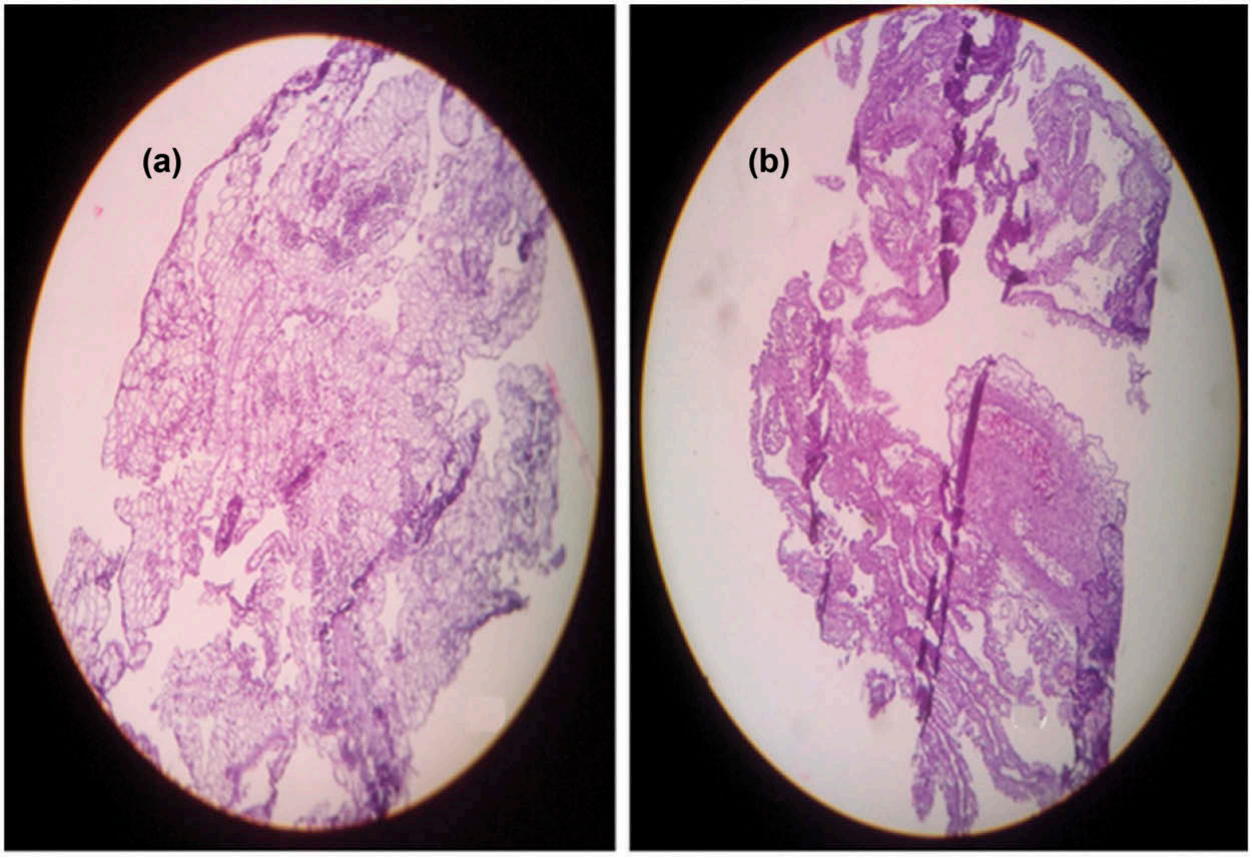
H and E staining of CAM (a) Control. (b) Extract treated group showed damage to the ectoderm layer compared to normal (40X magnifications).

*In vivo* antiangiogenic activity of the fungal extract was evaluated using chorioallantoic membrane (CAM) assay. *C. cladosporioides extract* treated CAM had (09.0 ± 0.37) less number of blood vessels than the control (18.0 ± 0.90).The decreases in the number of blood vessel branches on CAM confirmed the antiangiogenic potential of the extract (Figure 4). Morphological analysis of the *C. cladosporioides* extract treated CAM sections were carried out using haematoxylin and eosin staining. Hemetoxylin and eosin staining of the control and treated group of CAM showed a significant variation in the histomorphology. The control group had a normal organisation of CAM layers (ectoderm (ET), mesoderm (M) and endoderm (ED)), while endophytic extract treated CAM showed the damages to the ectodermal layer compared to control (Figure 5).

### Wound healing assay (in vitro scratch assay) by using MCF-7 cells

**Figure 6.**
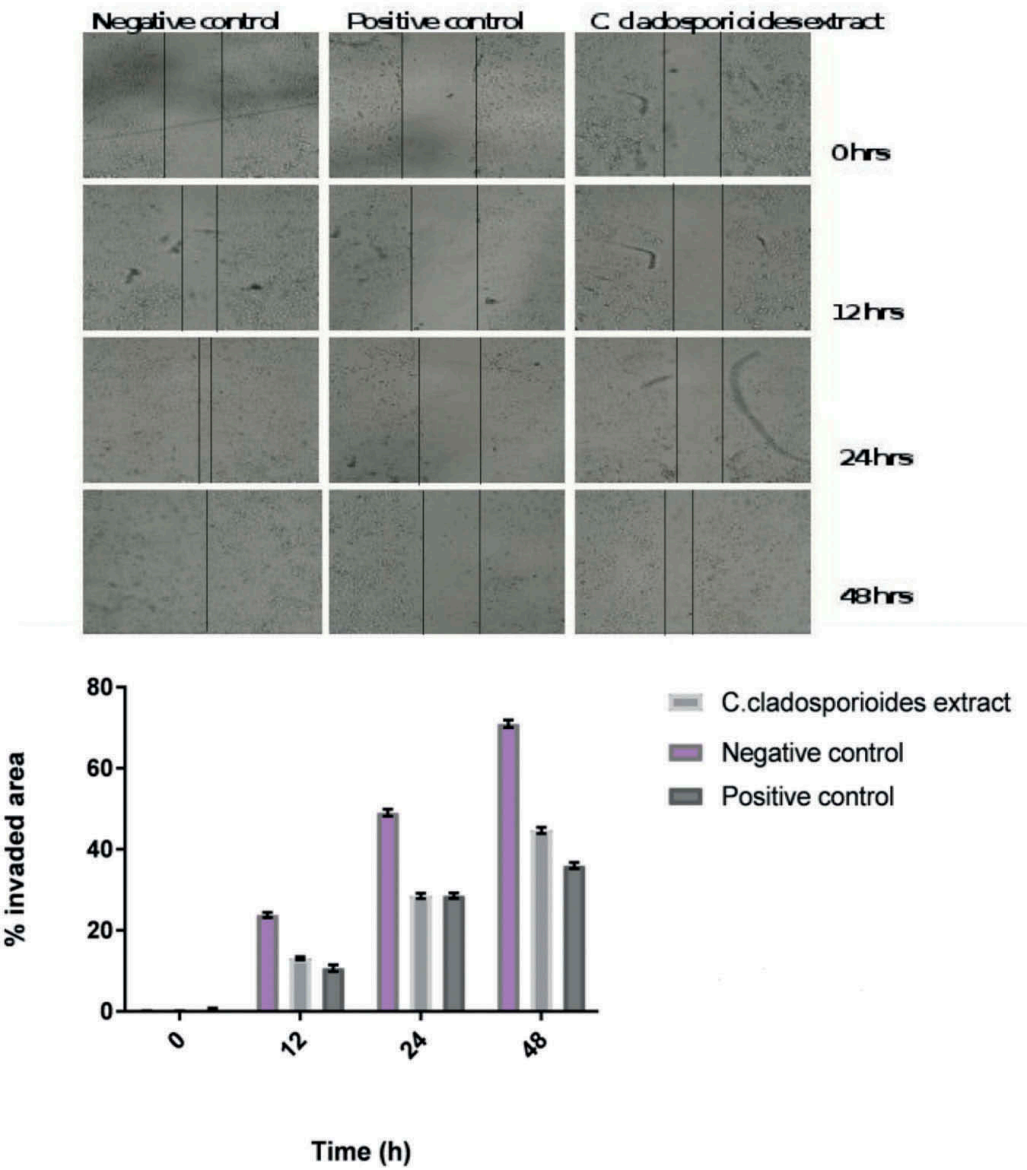
Scratch assay of MCF-7 cells showed a delayed process of wound healing. (a) Images of positive control Thalidomide 1 µg/ml, negative control and *C. cladosporioides* extract 1 mg/mL treated MCF-7 cells were taken at 0, 12 and 24 h. (b) Graphical representation of wound healing in positive control Thalidomide 1 µg/ml, negative control and *C. cladosporioides* extract 1 mg ⁄ mL treated MCF-7 cells at 0, 12 and 24 h.

MCF-7 cells with and without administration of *C. cladosporioides* extract were photographed at 0, 12 and 24 h. Scratch assay of MCF-7 cells showed a delayed process of wound healing. The MCF-7 cells treated with endophytic extract showed a slower rate of wound closure than the negative control cells and the extent of prevention of wound healing was comparable to the standard drug thalidomide (Figure 6).

### LC-MS analysis

**Figure 7.**
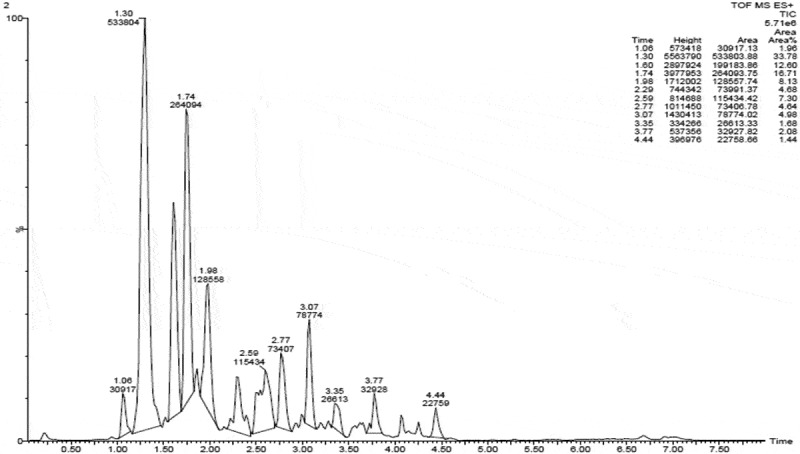
LC-MS analysis of ethyl acetate extract of *C. cladosporioides.*

The ethyl acetate extract of *C. cladosporioides* was subjected to LC-MS analysis to confirm the presence of different secondary metabolites. *C. cladosporioides* extract showed the presence of phenolic compounds in major amount (Figure 7, supplemental data). Comparison of molecular weights of the compounds present in the endophytic extract with the chemical database revealed the presence of N-(2-iodophenyl)-2-[2-oxo-5-(thiophen-2-yl)-2,3-dihydro-1,3,4-oxadiazol-3-yl]acetamide, 2-[3-chloro-4-(4-chlorophenoxy)phenyl]-1,3-dioxo-2,3-dihydro-1H-isoindole-5-carboxylic acid, 2-(2,4-dichlorophenyl)-2-oxoethyl 3,4-dihydro-2H-1,5-benzodioxepine-7-carboxylate, 4-bromo-N′-(4-fluoro-1-benzothiophene-2-carbonyl)-1H-pyrrole-2-carbohydrazide, 6-bromo-N-methyl-N-[2-(pyridin-4-yl)ethyl]-2-(trifluoromethyl)quinazolin-4-amine, (1R,2R,5S)-2-[3-({2-[(2,4-dichlorophenyl)methyl]-2H-1,2,3,4-tetrazol-5-yl}methyl)-4-methyl-5 sulfanylidene-4,5-dihydro-1H-1,2,4-triazol-1-yl]-6,8-dioxabicyclo[3.2.1]octan-4-one, methyl 2-({[5-bromo-2-(4 methoxybenzamido)phenyl](phenyl)methyl}amino)acetate, 2-[4-(2,4-dichlorophenoxy)phenyl]-5-phenyl-octahydro-1H-isoindole-1,3-dione,N-({2-[(3,4-dichlorophenyl)methoxy]naphthalen-1-yl}methyl)-2,3-dihydro-1,4-benzodioxin-6-amine, 2-({[(2,4-dichlorophenyl)carbamoyl]methyl}(propyl)amino)-N-(2,2,2-trifluoroethyl)acetamide, and N-(4-bromo-2-fluorophenyl)-6-(2-tert-butylhydrazin-1-yl)-5-nitropyrimidin-4-amine and N-(2-{3-[(3,4-dichlorophenyl)methyl]-2-oxo-1,3-diazinan-1-yl}-5-methylphenyl)-2,4-difluorobenzamide.

## Discussion

Novel bioactive secondary metabolites from marine microbes, especially from fungi, have been lately been studied for the development of principal therapeutic molecules against various diseases (Gao et al. 2011). The present study is the continuation of our recent report on the evaluation of the therapeutic potential of the endophytic fungus, *C. cladosporioides* isolated from the seaweed, *S. wightii* (Manjunath et al. 2016).

The antioxidant activity of endophytic extract was determined by DPPH radical scavenging activity. DPPH scavenging assay is one of the widely used methods to assess the antioxidant potential of natural products. This method is based on the reduction of purple coloured DPPH to a colourless solution as the natural products donate the electrons to DPPH free radicals (Brand-Williams et al. 1995). The DPPH radical scavenging activity of *C. cladosporioides* extract was much higher (Figure 3) than the activity observed in *P. citrinum* extract reported earlier (Manjunath et al. 2016). The different level of activities of the two fungi isolated from the same species may be attributed to the different chemical composition of each extract. This is in agreement with the observations of Gul et al. (2013).

The *C. cladosporioides* extract also showed significant Fe^3^^+^ reducing power. According to Gulcin (2006), electron donation capacity and free radical quenching capability are the hall marks of reducing power assay. The significant reducing power of the fungal extract can be attributed to the electron donation by the components present in it. Our study also highlighted the relationship between phenolic and flavonoid content to the antioxidant nature of the natural products. Our study is in agreement with the studies reported earlier about the positive correlation between the phenolic content and the antioxidant potential of natural products (Ben et al. 2009; Yadav et al. 2014; Campos et al. 2015).

Angiogenesis is essential for the tumour growth and metastasis as the process provides necessary oxygen and nutrition for the growing tumour. Antiangiogenic therapy and neovascularisation inhibition is a promising approach to the treatment of cancer. The CAM assay was carried out to verify the antiangiogenic activity of the endophytic extract *in vivo*. Angiogenesis on the chick CAM of fertilised eggs is the basis for CAM assay. CAM assay is frequently used to find out the angiogenic as well as the angiosuppressive potential of various molecules (Ribatti et al. 2001). In our present study, ethyl acetate extract of *C. cladosporioides* was subjected to its angiosuppressive activity in CAM assay. There was an increase in the vessel numbers and branches in the control group. However, a reduced length and number of vessel branches were observed in the *C. cladosporioides* extract treated group (Figure 4). Even avascular regions were observed around the insert in the treated group showing the suppression of angiogenesis on CAM. Hemetoxylin and eosin staining of the control and the treated group of CAM also confirmed the antiangiogenic capabilities of endophytic extract (Figure 5). Even though marine endophytes are studied for antioxidant, cytotoxic and pharmacological potential; much information was not available for the antiangiogenic activity. *C. cladosporioides* extract showed similar results as that of another fungus (*P.citrinum*) isolated from *S. weightii* as well as the activity of terrestrial and seaweed endophytes reported earlier (Ben et al. 2009; Udagawa et al. 2000; Namvar et al. 2013; Manjunath et al. 2016).

Administration of *C. cladosporioides* extract (1 mg/ml) and positive control (thalidomide, 1 µg/mL) showed a delayed process of wound closure (or invaded area) as shown in Figure 6 compared to the untreated MCF-7 cells. According to Hulkower and Herber (2011), key physiological and pathological process such as wound healing and cancer could be altered through the moderation cell migration as well as invasion. The wound healing potential of *C. cladosporioides* extract was much lower than thalidomide and other purified compound from the terrestrial endophytes. However, the purification of the lead molecule may show significant wound healing as that of standard drug (as we had used the crude extract for our study) This study is in agreement with the report of Manjunath et al. (2016).

The preliminary phytochemical tests and the LC-MS analysis of ethyl acetate extract of *C. cladosporioides* showed the presence of an array of secondary metabolites including phenolics (Figure 6).Phenolic compounds are reported to be responsible for reducing the lipid peroxidation and other types of oxidative process that leads to the generation of free radicals (Gulcin 2006; Yadav et al. 2014). According to Hassan et al. (2014) the extracts showing significant antioxidant activity are good antiangiogenic agents. The potential antiangiogenic activity of this endophytic extract may be attributed its antioxidant capacity and in turn to the phenolics and flavonoids.

We have reported for the first time about the potential antioxidant and antiangiogenic activity of ethyl extract of *C.cladosporoides*. Studies are going on in our lab to find out the active principle responsible for medicinal properties of this fungal extract. *Cladosporioides* extract could be used as a source of novel and cost-effective angiogenic modulators.

## Supplemental data

The supplemental data for this article can be accessed here
