# Supplementary material for: Antiangiogenic, wound healing and antioxidant activity of Cladosporium cladosporioides (Endophytic Fungus) isolated from seaweed (Sargassum wightii)
Source: Mycology. 2016 Dec 14;7(4):203–11. doi: 10.1080/21501203.2016.1263688 (PMC6059081; doi:10.1080/21501203.2016.1263688)
Supplement: supplementary_material.docx [file TMYC_A_1263688_SM5348.docx]

**Fig. 1** MS analysis of ethyl acetate extra ct of *C. Cladosporioides.*
